# Microscopical Investigations

**Published:** 1864-03

**Authors:** 


MICROSCOPICAL INVESTIGATIONS.—" One word of advice to those who by-and-bye intend entering upon regular medical studies. Familiarize yourselves with the use of the microscope, and in preserving the objects which you have examined. You want only a little glycerin, dilute acetic acid, and creosote water for preservative fluids, and then, with the help of a fragment of thin glass and a little Brunswick black, you may seal the object under examination upon a glass slide, and keep it by you for reference. You soon come to know which preservative fluid suits best. The object to be attained is an air-tight cell. Do not be over careful about appearances at first; neatness will attend experience. If you do not so far master the instrument before attendance upon lectures is begun, there will be no time for learning to do so during your studentship. You will doubtless be then anxious to examine the tissues as you read up their description, but you may find that your inexperience in the use of the instrument causes the expenditure of more time than you can afford, and the microscope is put by until, as you say, 'I have more time at my disposal.' But the little difficulties which you were unable to overcome in your busy moments grow by lapse of time to such an imaginary bulk as may, perhaps, ever after discourage you from resuming the use of an instrument which, in experienced hands, remains a fund of inexhaustible pleasure and instruction."—*Medical Times and Gazette.*

